# Supplementary figures and images for: Microvesicles from quiescent and TGF-β1 stimulated hepatic stellate cells: Divergent impact on hepatic vascular injury
Source: PLoS One. 2024 Jul 10;19(7):e0306775. doi: 10.1371/journal.pone.0306775 (PMC11236151; doi:10.1371/journal.pone.0306775)

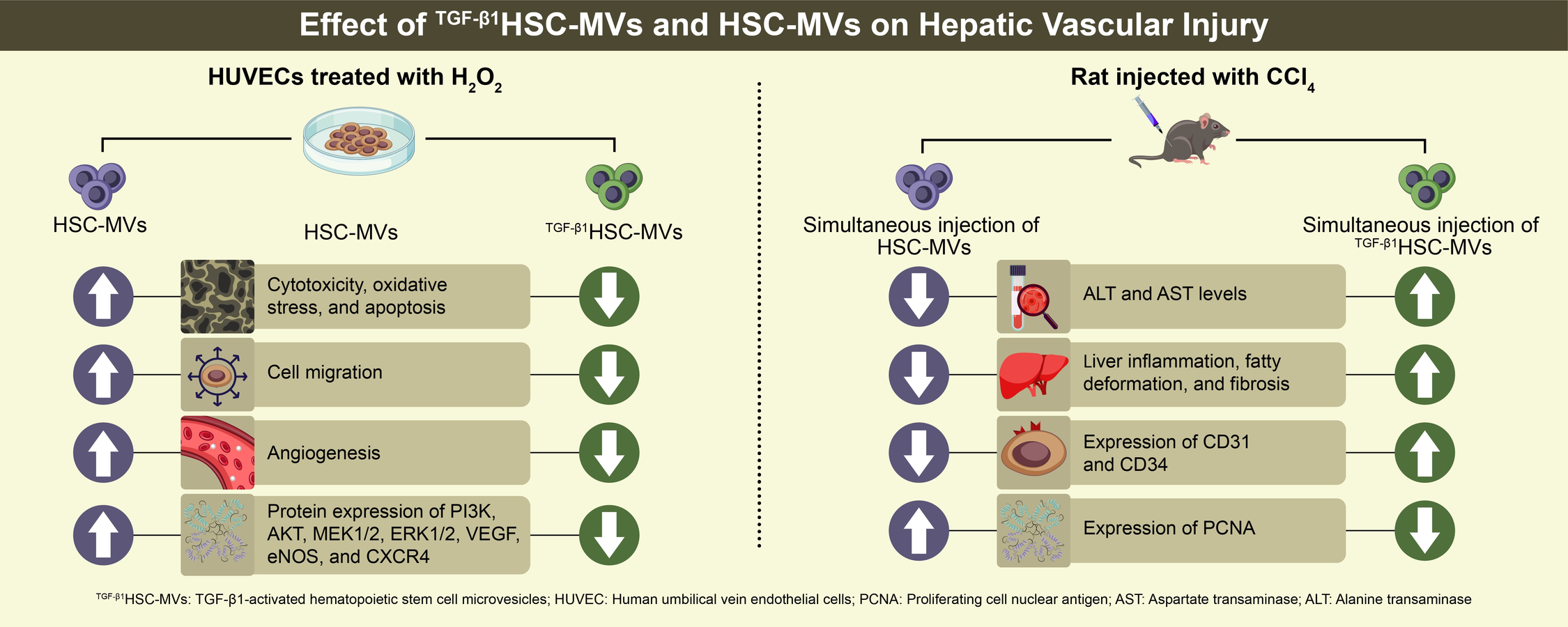

Supplement: S1 Graphical abstract — (TIF) [file pone.0306775.s001.tif]

Manipulated image

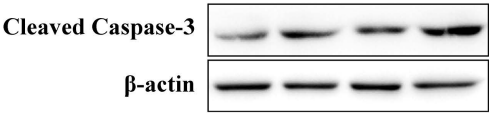

original image

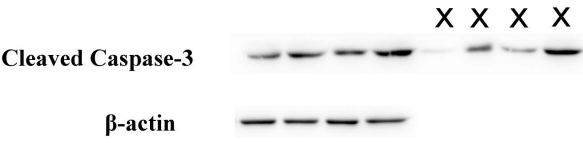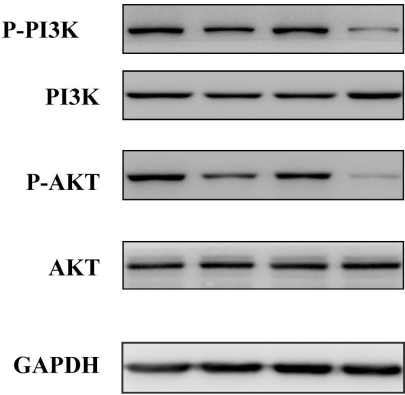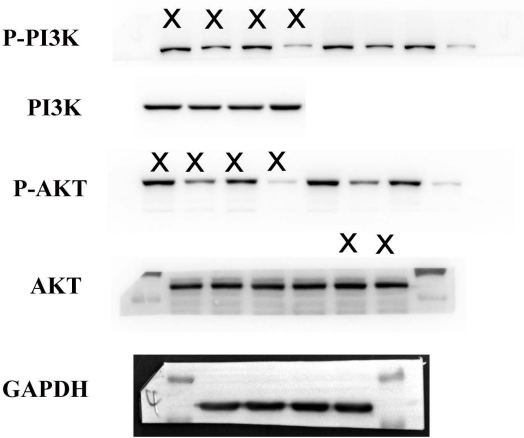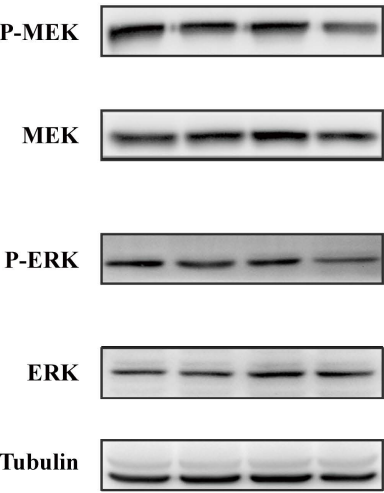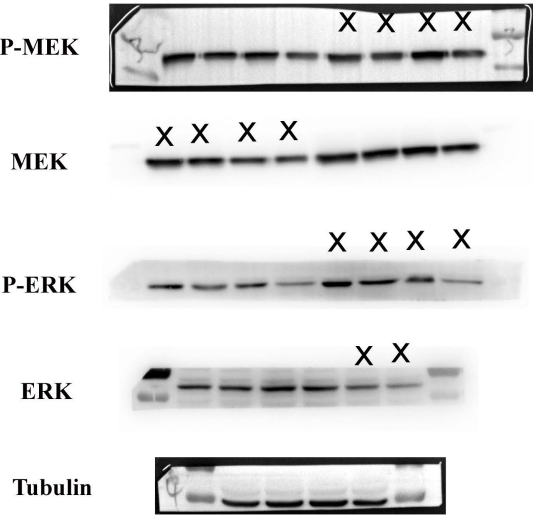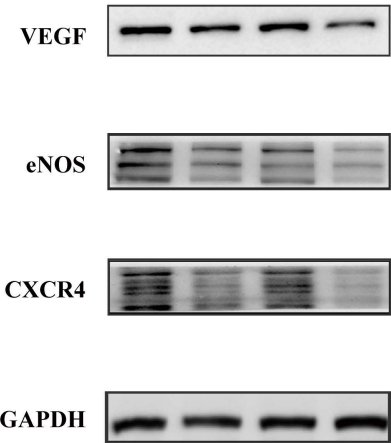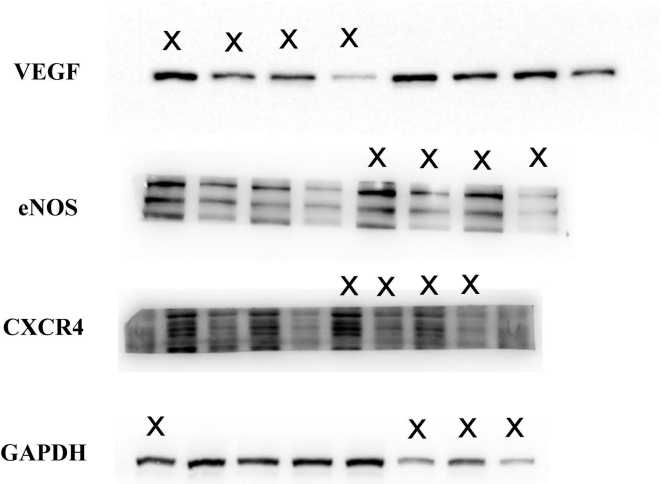

Supplement: S1 Raw images — (PDF) [file pone.0306775.s002.pdf]
