# Supplementary material for: Microvesicles from quiescent and TGF-β1 stimulated hepatic stellate cells: Divergent impact on hepatic vascular injury
Source: PLoS One. 2024 Jul 10;19(7):e0306775. doi: 10.1371/journal.pone.0306775 (PMC11236151; doi:10.1371/journal.pone.0306775)
Supplement: S1 Data — (PDF) [file pone.0306775.s003.pdf]

Original data in the manuscript

1. Figure 1

1-1. Figure 1B

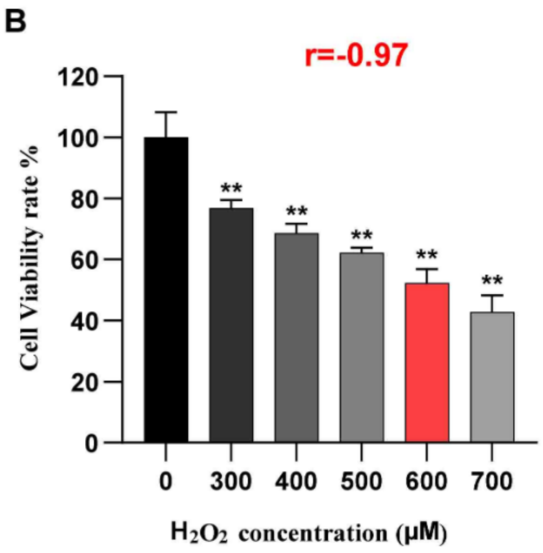

Descriptives

VAR00002

	N	Mean	Std. Deviation	Std. Error	95% Confidence Interval for Mean		Minimum	Maximum
					Lower Bound	Upper Bound		
Control	6	100.00000	8.275145	3.378314	91.31577	108.68423	89.271	110.440
300μmol/L H2O2	6	76.86152	2.556891	1.043847	74.17823	79.54482	73.942	80.416
400μmol/L H2O2	6	68.54529	3.243099	1.323990	65.14186	71.94871	64.965	73.510
500μmol/L H2O2	6	62.28124	1.524443	.622351	60.68144	63.88105	59.834	64.333
600μmol/L H2O2	6	52.34806	4.579297	1.869490	47.54239	57.15374	46.842	58.837
700μmol/L H2O2	6	42.86549	5.454038	2.226602	37.14183	48.58916	35.471	50.118
Total	36	67.15027	19.079086	3.179848	60.69483	73.60570	35.471	110.440

ANOVA

VAR00002

	Sum of Squares	df	Mean Square	F	Sig.
Between Groups	12047.534	5	2409.507	104.327	.000
Within Groups	692.869	30	23.096		
Total	12740.403	35			

Post Hoc Tests

Multiple Comparisons

Dependent Variable: VAR00002
LSD

(I) VAR00001	(J) VAR00001	Mean Difference (I-J)	Std. Error	Sig.	95% Confidence Interval	
					Lower Bound	Upper Bound
Control	300μmol/L H2O2	23.138475*	2.774625	.000	17.47193	28.80502
	400μmol/L H2O2	31.454714*	2.774625	.000	25.78817	37.12125
	500μmol/L H2O2	37.718759*	2.774625	.000	32.05222	43.38530
	600μmol/L H2O2	47.651936*	2.774625	.000	41.98539	53.31848
	700μmol/L H2O2	57.134506*	2.774625	.000	51.46797	62.80105
300μmol/L H2O2	Control	-23.138475*	2.774625	.000	-28.80502	-17.47193
	400μmol/L H2O2	8.316239*	2.774625	.005	2.64970	13.98278
	500μmol/L H2O2	14.580284*	2.774625	.000	8.91374	20.24682
	600μmol/L H2O2	24.513460*	2.774625	.000	18.84692	30.18000
	700μmol/L H2O2	33.996031*	2.774625	.000	28.32949	39.66257
400μmol/L H2O2	Control	-31.454714*	2.774625	.000	-37.12125	-25.78817
	300μmol/L H2O2	-8.316239*	2.774625	.005	-13.98278	-2.64970
	500μmol/L H2O2	6.264045*	2.774625	.031	.59750	11.93059
	600μmol/L H2O2	16.197222*	2.774625	.000	10.53068	21.86376
	700μmol/L H2O2	25.679792*	2.774625	.000	20.01325	31.34633
500μmol/L H2O2	Control	-37.718759*	2.774625	.000	-43.38530	-32.05222
	300μmol/L H2O2	-14.580284*	2.774625	.000	-20.24682	-8.91374
	400μmol/L H2O2	-6.264045*	2.774625	.031	-11.93059	-.59750
	600μmol/L H2O2	9.933176*	2.774625	.001	4.26664	15.59972
	700μmol/L H2O2	19.415747*	2.774625	.000	13.74921	25.08229
600μmol/L H2O2	Control	-47.651936*	2.774625	.000	-53.31848	-41.98539
	300μmol/L H2O2	-24.513460*	2.774625	.000	-30.18000	-18.84692
	400μmol/L H2O2	-16.197222*	2.774625	.000	-21.86376	-10.53068
	500μmol/L H2O2	-9.933176*	2.774625	.001	-15.59972	-4.26664
	700μmol/L H2O2	9.482570*	2.774625	.002	3.81603	15.14911
700μmol/L H2O2	Control	-57.134506*	2.774625	.000	-62.80105	-51.46797
	300μmol/L H2O2	-33.996031*	2.774625	.000	-39.66257	-28.32949
	400μmol/L H2O2	-25.679792*	2.774625	.000	-31.34633	-20.01325
	500μmol/L H2O2	-19.415747*	2.774625	.000	-25.08229	-13.74921
	600μmol/L H2O2	-9.482570*	2.774625	.002	-15.14911	-3.81603

*. The mean difference is significant at the 0.05 level.

1-2. Figure 1C

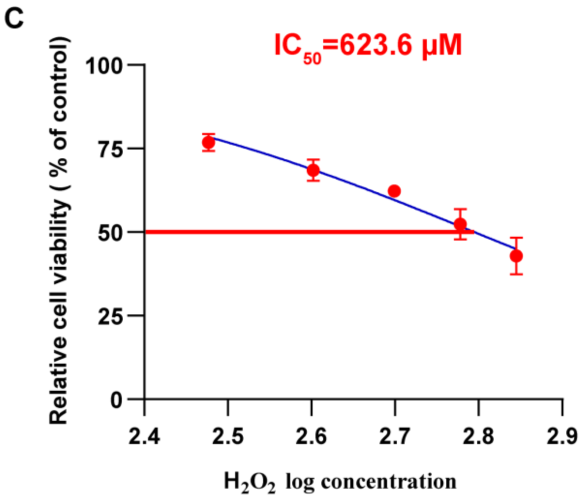

R=-.971

Descriptive Statistics

	Mean	Std. Deviation	N
VAR00002	416.66667	229.906813	36
VAR00003	67.1503	19.07909	36

Correlations

		VAR00002	VAR00003
VAR00002	Pearson Correlation	1	-.971**
	Sig. (2-tailed)		.000
	N	36	36
VAR00003	Pearson Correlation	-.971**	1
	Sig. (2-tailed)	.000	
	N	36	36

**. Correlation is significant at the 0.01 level (2-tailed).

1-1. Figure 1C

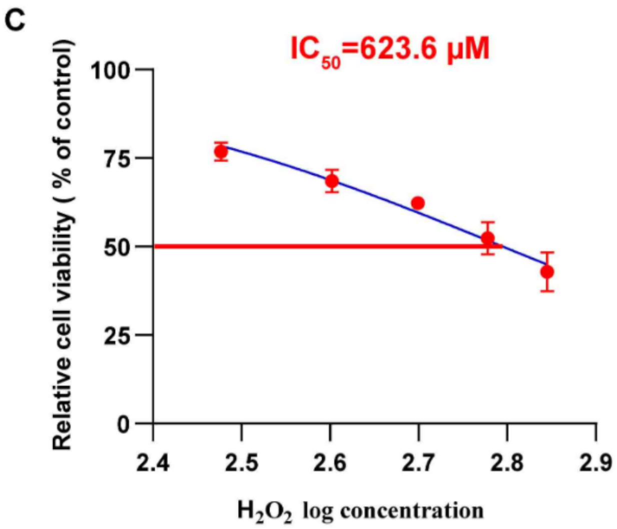

	Nonlin fit<br>Table of results	A<br>Cell viability rate %	B
1	**log(inhibitor) vs. normalized response -- Variable slope**		
2	**Best-fit values**		
3	LogIC50	2.795	
4	HillSlope	-1.765	
5	IC50	623.6	
6	**95% CI (profile likelihood)**		
7	LogIC50	2.777 to 2.816	
8	HillSlope	-2.015 to -1.525	
9	IC50	597.9 to 655.3	
10	**Goodness of Fit**		
11	Degrees of Freedom	28	
12	R squared	0.9059	
13	Sum of Squares	435.6	
14	Sy.x	3.944	
15			
16	**Number of points**		
17	# of X values	30	
18	# Y values analyzed	30	
19			

2. Figure 2

2-1. Figure 2A

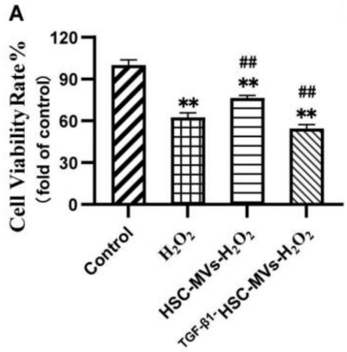

Descriptives

VAR00002

	N	Mean	Std. Deviation	Std. Error	95% Confidence Interval for Mean		Minimum	Maximum
					Lower Bound	Upper Bound		
Control	4	100.00000	3.828762	1.914381	93.90759	106.09241	95.806	104.795
H2O2	4	62.54496	3.194512	1.597256	57.46178	67.62814	58.736	66.269
HSC-MVs-H2O2	4	76.36156	1.882842	.941421	73.36554	79.35758	73.971	78.372
TGF-β1-HSC-MVs-H2O2	4	54.50468	2.758101	1.379050	50.11592	58.89343	51.627	57.721
Total	16	73.35280	18.023684	4.505921	63.74866	82.95694	51.627	104.795

ANOVA

VAR00002

	Sum of Squares	df	Mean Square	F	Sig.
Between Groups	4764.748	3	1588.249	176.391	.000
Within Groups	108.050	12	9.004		
Total	4872.798	15			

Post Hoc Tests

Multiple Comparisons

Dependent Variable: VAR00002
LSD

(I) VAR00001	(J) VAR00001	Mean Difference (I-J)	Std. Error	Sig.	95% Confidence Interval	
					Lower Bound	Upper Bound
Control	H2O2	37.455038*	2.121808	.000	32.83202	42.07806
	HSC-MVs-H2O2	23.638441*	2.121808	.000	19.01542	28.26146
	TGF-β1-HSC-MVs-H2O2	45.495324*	2.121808	.000	40.87230	50.11835
H2O2	Control	-37.455038*	2.121808	.000	-42.07806	-32.83202
	HSC-MVs-H2O2	-13.816597*	2.121808	.000	-18.43962	-9.19358
	TGF-β1-HSC-MVs-H2O2	8.040286*	2.121808	.003	3.41726	12.66331
HSC-MVs-H2O2	Control	-23.638441*	2.121808	.000	-28.26146	-19.01542
	H2O2	13.816597*	2.121808	.000	9.19358	18.43962
	TGF-β1-HSC-MVs-H2O2	21.856883*	2.121808	.000	17.23386	26.47990
TGF-β1-HSC-MVs-H2O2	Control	-45.495324*	2.121808	.000	-50.11835	-40.87230
	H2O2	-8.040286*	2.121808	.003	-12.66331	-3.41726
	HSC-MVs-H2O2	-21.856883*	2.121808	.000	-26.47990	-17.23386

*. The mean difference is significant at the 0.05 level.

2-2. Figure 2B

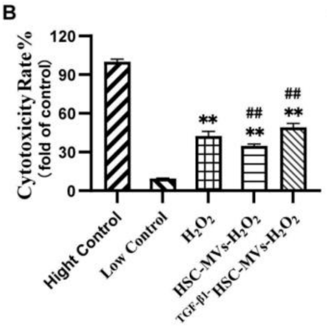

Descriptives

VAR00002

	N	Mean	Std. Deviation	Std. Error	95% Confidence Interval for Mean		Minimum	Maximum
					Lower Bound	Upper Bound		
High Control	4	100.00000	2.147209	1.073604	96.58331	103.41669	97.215	102.418
Low Control	4	9.39121	.394668	.197334	8.76320	10.01921	8.904	9.798
H2O2	4	42.49581	3.459928	1.729964	36.99029	48.00133	38.937	46.118
HSC-MVs-H2O2	4	34.74826	1.513981	.756990	32.33918	37.15735	33.033	36.575
TGF-β1-HSC-MVs-H2O2	4	49.26993	3.039028	1.519514	44.43415	54.10570	45.129	51.704
Total	20	47.18104	30.504535	6.821021	32.90448	61.45760	8.904	102.418

ANOVA

VAR00002

	Sum of Squares	df	Mean Square	F	Sig.
Between Groups	17595.211	4	4398.803	778.131	.000
Within Groups	84.796	15	5.653		
Total	17680.006	19			

Post Hoc Tests

Multiple Comparisons

Dependent Variable: VAR00002
LSD

(I) VAR00001	(J) VAR00001	Mean Difference (I-J)	Std. Error	Sig.	95% Confidence Interval	
					Lower Bound	Upper Bound
High Control	Low Control	90.608793*	1.681226	.000	87.02535	94.19224
	H2O2	57.504189*	1.681226	.000	53.92074	61.08764
	HSC-MVs-H2O2	65.251735*	1.681226	.000	61.66829	68.83518
	TGF-β1-HSC-MVs-H2O2	50.730073*	1.681226	.000	47.14662	54.31352
Low Control	High Control	-90.608793*	1.681226	.000	-94.19224	-87.02535
	H2O2	-33.104604*	1.681226	.000	-36.68805	-29.52116
	HSC-MVs-H2O2	-25.357057*	1.681226	.000	-28.94051	-21.77361
	TGF-β1-HSC-MVs-H2O2	-39.878720*	1.681226	.000	-43.46217	-36.29527
H2O2	High Control	-57.504189*	1.681226	.000	-61.08764	-53.92074
	Low Control	33.104604*	1.681226	.000	29.52116	36.68805
	HSC-MVs-H2O2	7.747546*	1.681226	.000	4.16410	11.33099
	TGF-β1-HSC-MVs-H2O2	-6.774116*	1.681226	.001	-10.35756	-3.19067
HSC-MVs-H2O2	High Control	-65.251735*	1.681226	.000	-68.83518	-61.66829
	Low Control	25.357057*	1.681226	.000	21.77361	28.94051
	H2O2	-7.747546*	1.681226	.000	-11.33099	-4.16410
	TGF-β1-HSC-MVs-H2O2	-14.521663*	1.681226	.000	-18.10511	-10.93822
TGF-β1-HSC-MVs-H2O2	High Control	-50.730073*	1.681226	.000	-54.31352	-47.14662
	Low Control	39.878720*	1.681226	.000	36.29527	43.46217
	H2O2	6.774116*	1.681226	.001	3.19067	10.35756
	HSC-MVs-H2O2	14.521663*	1.681226	.000	10.93822	18.10511

*. The mean difference is significant at the 0.05 level.

2-3. Figure 2C

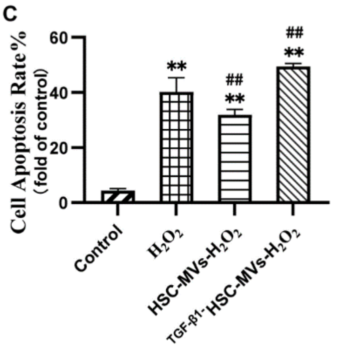

Descriptives

VAR00002

	N	Mean	Std. Deviation	Std. Error	95% Confidence Interval for Mean		Minimum	Maximum
					Lower Bound	Upper Bound		
Control	3	4.3333	.77675	.44845	2.4038	6.2629	3.70	5.20
H2O2	3	40.1667	5.17333	2.98682	27.3154	53.0179	34.20	43.40
HSC-MVs-H2O2	3	31.8333	2.00333	1.15662	26.8568	36.8099	30.30	34.10
TGF-β1-HSC-MVs-H2O2	3	49.4333	1.05987	.61192	46.8005	52.0662	48.30	50.40
Total	12	31.4417	17.75984	5.12682	20.1576	42.7257	3.70	50.40

ANOVA

VAR00002

	Sum of Squares	df	Mean Square	F	Sig.
Between Groups	3404.522	3	1134.841	139.658	.000
Within Groups	65.007	8	8.126		
Total	3469.529	11			

Post Hoc Tests

Multiple Comparisons

Dependent Variable: VAR00002
LSD

(I) VAR00001	(J) VAR00001	Mean Difference (I-J)	Std. Error	Sig.	95% Confidence Interval	
					Lower Bound	Upper Bound
Control	H2O2	-35.83333*	2.32749	.000	-41.2005	-30.4661
	HSC-MVs-H2O2	-27.50000*	2.32749	.000	-32.8672	-22.1328
	TGF-β1-HSC-MVs-H2O2	-45.10000*	2.32749	.000	-50.4672	-39.7328
H2O2	Control	35.83333*	2.32749	.000	30.4661	41.2005
	HSC-MVs-H2O2	8.33333*	2.32749	.007	2.9661	13.7005
	TGF-β1-HSC-MVs-H2O2	-9.26667*	2.32749	.004	-14.6339	-3.8995
HSC-MVs-H2O2	Control	27.50000*	2.32749	.000	22.1328	32.8672
	H2O2	-8.33333*	2.32749	.007	-13.7005	-2.9661
	TGF-β1-HSC-MVs-H2O2	-17.60000*	2.32749	.000	-22.9672	-12.2328
TGF-β1-HSC-MVs-H2O2	Control	45.10000*	2.32749	.000	39.7328	50.4672
	H2O2	9.26667*	2.32749	.004	3.8995	14.6339
	HSC-MVs-H2O2	17.60000*	2.32749	.000	12.2328	22.9672

*. The mean difference is significant at the 0.05 level.

2-3. Figure 2F

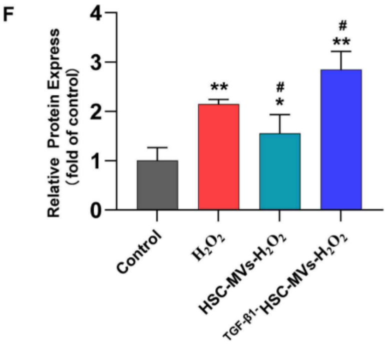

Descriptives

VAR00002

	N	Mean	Std. Deviation	Std. Error	95% Confidence Interval for Mean Lower Bound	95% Confidence Interval for Mean Upper Bound	Minimum	Maximum
Control	3	1.00211	.265552	.153317	.34244	1.66178	.700	1.199
H2O2	3	2.14735	.096520	.055726	1.90758	2.38711	2.053	2.246
HSC-MVs-H2O2	3	1.55467	.379581	.219151	.61174	2.49760	1.132	1.867
TGF-β1-HSC-MVs-H2O2	3	2.84473	.369123	.213113	1.92778	3.76168	2.429	3.135
Total	12	1.88721	.760134	.219432	1.40425	2.37018	.700	3.135

ANOVA

VAR00002

	Sum of Squares	df	Mean Square	F	Sig.
Between Groups	5.636	3	1.879	20.863	.000
Within Groups	.720	8	.090		
Total	6.356	11			

Post Hoc Tests

Multiple Comparisons

Dependent Variable: VAR00002
LSD

(I) VAR00001	(J) VAR00001	Mean Difference (I-J)	Std. Error	Sig.	95% Confidence Interval Lower Bound	95% Confidence Interval Upper Bound
Control	H2O2	-1.145238*	.245006	.002	-1.71022	-.58025
	HSC-MVs-H2O2	-.552562	.245006	.054	-1.11755	.01242
	TGF-β1-HSC-MVs-H2O2	-1.842621*	.245006	.000	-2.40761	-1.27764
H2O2	Control	1.145238*	.245006	.002	.58025	1.71022
	HSC-MVs-H2O2	.592676*	.245006	.042	.02769	1.15766
	TGF-β1-HSC-MVs-H2O2	-.697383*	.245006	.022	-1.26237	-.13240
HSC-MVs-H2O2	Control	.552562	.245006	.054	-.01242	1.11755
	H2O2	-.592676*	.245006	.042	-1.15766	-.02769
	TGF-β1-HSC-MVs-H2O2	-1.290059*	.245006	.001	-1.85504	-.72507
TGF-β1-HSC-MVs-H2O2	Control	1.842621*	.245006	.000	1.27764	2.40761
	H2O2	.697383*	.245006	.022	.13240	1.26237
	HSC-MVs-H2O2	1.290059*	.245006	.001	.72507	1.85504

*. The mean difference is significant at the 0.05 level.

3. Figure 3

3-1. Figure 3D

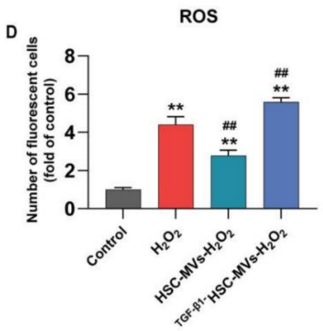

Descriptives

VAR00002

	N	Mean	Std. Deviation	Std. Error	95% Confidence Interval for Mean Lower Bound	95% Confidence Interval for Mean Upper Bound	Minimum	Maximum
Control	4	1.00000	.100413	.050206	.84022	1.15978	.902	1.123
H2O2	4	4.40250	.417052	.208526	3.73888	5.06613	3.964	4.937
HSC-MVs-H2O2	4	2.77818	.275678	.137839	2.33951	3.21684	2.476	3.077
TGF-β1-HSC-MVs-H2O2	4	5.58676	.216835	.108417	5.24173	5.93179	5.395	5.868
Total	16	3.44186	1.800509	.450127	2.48244	4.40128	.902	5.868

ANOVA

VAR00002

	Sum of Squares	df	Mean Square	F	Sig.
Between Groups	47.706	3	15.902	207.173	.000
Within Groups	.921	12	.077		
Total	48.627	15			

Post Hoc Tests

Multiple Comparisons

Dependent Variable: VAR00002
LSD

(I) VAR00001	(J) VAR00001	Mean Difference (I-J)	Std. Error	Sig.	95% Confidence Interval Lower Bound	95% Confidence Interval Upper Bound
Control	H2O2	-3.402504*	.195905	.000	-3.82935	-2.97566
	HSC-MVs-H2O2	-1.778175*	.195905	.000	-2.20502	-1.35133
	TGF-β1-HSC-MVs-H2O2	-4.586762*	.195905	.000	-5.01360	-4.15992
H2O2	Control	3.402504*	.195905	.000	2.97566	3.82935
	HSC-MVs-H2O2	1.624329*	.195905	.000	1.19749	2.05117
	TGF-β1-HSC-MVs-H2O2	-1.184258*	.195905	.000	-1.61110	-.75742
HSC-MVs-H2O2	Control	1.778175*	.195905	.000	1.35133	2.20502
	H2O2	-1.624329*	.195905	.000	-2.05117	-1.19749
	TGF-β1-HSC-MVs-H2O2	-2.808587*	.195905	.000	-3.23543	-2.38175
TGF-β1-HSC-MVs-H2O2	Control	4.586762*	.195905	.000	4.15992	5.01360
	H2O2	1.184258*	.195905	.000	.75742	1.61110
	HSC-MVs-H2O2	2.808587*	.195905	.000	2.38175	3.23543

*. The mean difference is significant at the 0.05 level.

3-2. Figure 3E

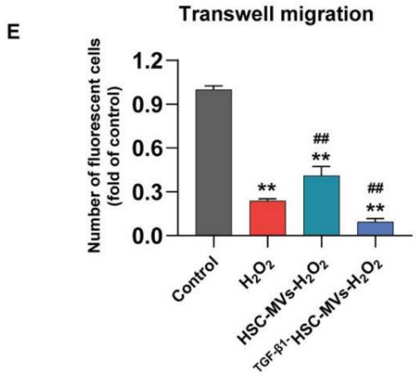

Descriptives

VAR00002

	N	Mean	Std. Deviation	Std. Error	95% Confidence Interval for Mean		Minimum	Maximum
					Lower Bound	Upper Bound		
Control	3	1.0000	.02499	.01443	.9379	1.0621	.98	1.03
H2O2	3	.2383	.01398	.00807	.2035	.2730	.22	.25
HSC-MVs-H2O2	3	.4100	.06370	.03678	.2517	.5682	.36	.48
TGF-β1-HSC-MVs-H2O2	3	.0940	.02235	.01290	.0385	.1495	.07	.12
Total	12	.4356	.36122	.10428	.2060	.6651	.07	1.03

ANOVA

VAR00002

	Sum of Squares	df	Mean Square	F	Sig.
Between Groups	1.425	3	.475	353.213	.000
Within Groups	.011	8	.001		
Total	1.435	11			

Post Hoc Tests

Multiple Comparisons

Dependent Variable: VAR00002
LSD

(I) VAR00001	(J) VAR00001	Mean Difference (I-J)	Std. Error	Sig.	95% Confidence Interval	
					Lower Bound	Upper Bound
Control	H2O2	.76175*	.02994	.000	.6927	.8308
	HSC-MVs-H2O2	.59004*	.02994	.000	.5210	.6591
	TGF-β1-HSC-MVs-H2O2	.90600*	.02994	.000	.8370	.9750
H2O2	Control	-.76175*	.02994	.000	-.8308	-.6927
	HSC-MVs-H2O2	-.17171*	.02994	.000	-.2407	-.1027
	TGF-β1-HSC-MVs-H2O2	.14425*	.02994	.001	.0752	.2133
HSC-MVs-H2O2	Control	-.59004*	.02994	.000	-.6591	-.5210
	H2O2	.17171*	.02994	.000	.1027	.2407
	TGF-β1-HSC-MVs-H2O2	.31596*	.02994	.000	.2469	.3850
TGF-β1-HSC-MVs-H2O2	Control	-.90600*	.02994	.000	-.9750	-.8370
	H2O2	-.14425*	.02994	.001	-.2133	-.0752
	HSC-MVs-H2O2	-.31596*	.02994	.000	-.3850	-.2469

*. The mean difference is significant at the 0.05 level.

3-3. Figure 3F

F

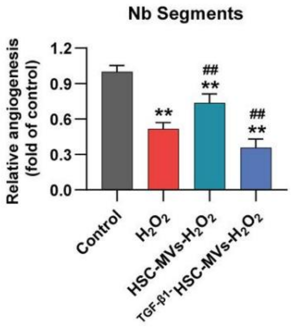

Descriptives

VAR00002

	N	Mean	Std. Deviation	Std. Error	95% Confidence Interval for Mean		Minimum	Maximum
					Lower Bound	Upper Bound		
Control	4	1.00000	.051391	.025695	.91823	1.08177	.959	1.074
H2O2	4	.51653	.053347	.026673	.43164	.60142	.455	.579
HSC-MVs-H2O2	4	.73554	.076938	.038469	.61311	.85796	.645	.826
TGF-β1-HSC-MVs-H2O2	4	.35744	.073417	.036709	.24061	.47426	.289	.455
Total	16	.65238	.256020	.064005	.51595	.78880	.289	1.074

ANOVA

VAR00002

	Sum of Squares	df	Mean Square	F	Sig.
Between Groups	.933	3	.311	74.048	.000
Within Groups	.050	12	.004		
Total	.983	15			

Post Hoc Tests

Multiple Comparisons

Dependent Variable: VAR00002

LSD

(I) VAR00001	(J) VAR00001	Mean Difference (I-J)	Std. Error	Sig.	95% Confidence Interval	
					Lower Bound	Upper Bound
Control	H2O2	.483471*	.045821	.000	.38364	.58331
	HSC-MVs-H2O2	.264463*	.045821	.000	.16463	.36430
	TGF-β1-HSC-MVs-H2O2	.642562*	.045821	.000	.54273	.74240
H2O2	Control	-.483471*	.045821	.000	-.58331	-.38364
	HSC-MVs-H2O2	-.219008*	.045821	.000	-.31884	-.11917
	TGF-β1-HSC-MVs-H2O2	.159091*	.045821	.005	.05926	.25893
HSC-MVs-H2O2	Control	-.264463*	.045821	.000	-.36430	-.16463
	H2O2	.219008*	.045821	.000	.11917	.31884
	TGF-β1-HSC-MVs-H2O2	.378099*	.045821	.000	.27826	.47793
TGF-β1-HSC-MVs-H2O2	Control	-.642562*	.045821	.000	-.74240	-.54273
	H2O2	-.159091*	.045821	.005	-.25893	-.05926
	HSC-MVs-H2O2	-.378099*	.045821	.000	-.47793	-.27826

*. The mean difference is significant at the 0.05 level.

3-4. Figure 3G

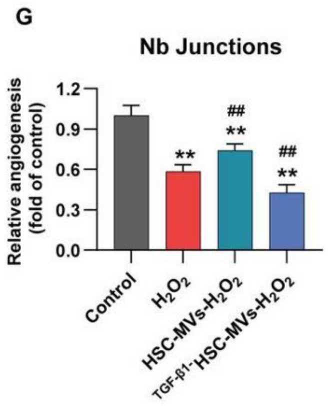

Descriptives

VAR00002

	N	Mean	Std. Deviation	Std. Error	95% Confidence Interval for Mean		Minimum	Maximum
					Lower Bound	Upper Bound		
Control	4	1.00000	.075332	.037666	.88013	1.11987	.918	1.091
H2O2	4	.58409	.052157	.026079	.50110	.66708	.536	.655
HSC-MVs-H2O2	4	.74091	.047817	.023909	.66482	.81700	.700	.809
TGF-β1-HSC-MVs-H2O2	4	.42727	.058916	.029458	.33352	.52102	.364	.500
Total	16	.68807	.224816	.056204	.56827	.80786	.364	1.091

ANOVA

VAR00002

	Sum of Squares	df	Mean Square	F	Sig.
Between Groups	.716	3	.239	67.423	.000
Within Groups	.042	12	.004		
Total	.758	15			

Post Hoc Tests

Multiple Comparisons

Dependent Variable: VAR00002
LSD

(I) VAR00001	(J) VAR00001	Mean Difference (I-J)	Std. Error	Sig.	95% Confidence Interval	
					Lower Bound	Upper Bound
Control	H2O2	.415909*	.042061	.000	.32427	.50755
	HSC-MVs-H2O2	.259091*	.042061	.000	.16745	.35073
	TGF-β1-HSC-MVs-H2O2	.572727*	.042061	.000	.48108	.66437
H2O2	Control	-.415909*	.042061	.000	-.50755	-.32427
	HSC-MVs-H2O2	-.156818*	.042061	.003	-.24846	-.06518
	TGF-β1-HSC-MVs-H2O2	.156818*	.042061	.003	.06518	.24846
HSC-MVs-H2O2	Control	-.259091*	.042061	.000	-.35073	-.16745
	H2O2	.156818*	.042061	.003	.06518	.24846
	TGF-β1-HSC-MVs-H2O2	.313636*	.042061	.000	.22199	.40528
TGF-β1-HSC-MVs-H2O2	Control	-.572727*	.042061	.000	-.66437	-.48108
	H2O2	-.156818*	.042061	.003	-.24846	-.06518
	HSC-MVs-H2O2	-.313636*	.042061	.000	-.40528	-.22199

*. The mean difference is significant at the 0.05 level.

3-5. Figure 3H

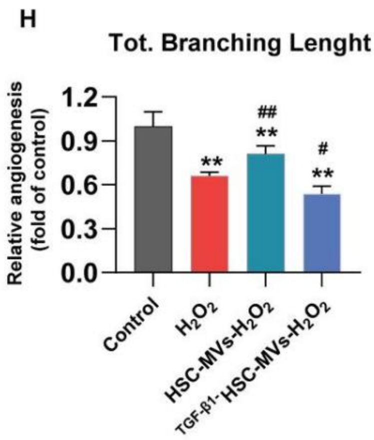

Descriptives

VAR00002

	N	Mean	Std. Deviation	Std. Error	95% Confidence Interval for Mean Lower Bound	95% Confidence Interval for Mean Upper Bound	Minimum	Maximum
Control	4	1.00000	.098679	.049339	.84298	1.15702	.883	1.117
H2O2	4	.66229	.023329	.011665	.62517	.69941	.633	.687
HSC-MVs-H2O2	4	.81072	.055064	.027532	.72310	.89834	.744	.876
TGF-β1-HSC-MVs-H2O2	4	.53868	.051570	.025785	.45662	.62074	.464	.579
Total	16	.75292	.186534	.046634	.65352	.85232	.464	1.117

ANOVA

VAR00002

	Sum of Squares	df	Mean Square	F	Sig.
Between Groups	.474	3	.158	39.567	.000
Within Groups	.048	12	.004		
Total	.522	15			

Post Hoc Tests

Multiple Comparisons

Dependent Variable: VAR00002
LSD

(I) VAR00001	(J) VAR00001	Mean Difference (I-J)	Std. Error	Sig.	95% Confidence Interval Lower Bound	95% Confidence Interval Upper Bound
Control	H2O2	.337710*	.044684	.000	.24035	.43507
	HSC-MVs-H2O2	.189282*	.044684	.001	.09192	.28664
	TGF-β1-HSC-MVs-H2O2	.461320*	.044684	.000	.36396	.55868
H2O2	Control	-.337710*	.044684	.000	-.43507	-.24035
	HSC-MVs-H2O2	-.148428*	.044684	.006	-.24579	-.05107
	TGF-β1-HSC-MVs-H2O2	.123610*	.044684	.017	.02625	.22097
HSC-MVs-H2O2	Control	-.189282*	.044684	.001	-.28664	-.09192
	H2O2	.148428*	.044684	.006	.05107	.24579
	TGF-β1-HSC-MVs-H2O2	.272037*	.044684	.000	.17468	.36940
TGF-β1-HSC-MVs-H2O2	Control	-.461320*	.044684	.000	-.55868	-.36396
	H2O2	-.123610*	.044684	.017	-.22097	-.02625
	HSC-MVs-H2O2	-.272037*	.044684	.000	-.36940	-.17468

*. The mean difference is significant at the 0.05 level.

4. Figure 4

4-1. Figure 4B

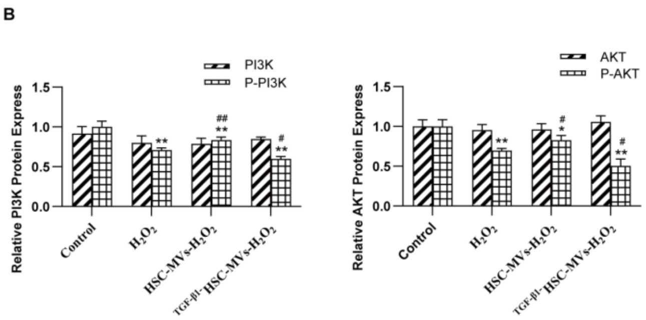

4-1-1. Figure 4B PI3K

Descriptives

VAR00002

	N	Mean	Std. Deviation	Std. Error	95% Confidence Interval for Mean Lower Bound	Upper Bound	Minimum	Maximum
Control	3	.9166	.08802	.05082	.6979	1.1352	.83	1.00
H2O2	3	.7983	.08970	.05179	.5755	1.0212	.70	.88
HSC-MVs-H2O2	3	.7875	.06985	.04033	.6140	.9610	.71	.85
TGF-β1-HSC-MVs-H2O2	3	.8476	.02488	.01437	.7858	.9094	.83	.88
Total	12	.8375	.08188	.02364	.7855	.8895	.70	1.00

ANOVA

VAR00002

	Sum of Squares	df	Mean Square	F	Sig.
Between Groups	.031	3	.010	1.951	.200
Within Groups	.043	8	.005		
Total	.074	11			

Post Hoc Tests

Multiple Comparisons

Dependent Variable: VAR00002
Tamhane

(I) VAR00001	(J) VAR00001	Mean Difference (I-J)	Std. Error	Sig.	95% Confidence Interval Lower Bound	Upper Bound
Control	H2O2	.11823	.07256	.693	-.2317	.4682
	HSC-MVs-H2O2	.12907	.06487	.539	-.1956	.4537
	TGF-β1-HSC-MVs-H2O2	.06897	.05281	.888	-.3801	.5181
H2O2	Control	-.11823	.07256	.693	-.4682	.2317
	HSC-MVs-H2O2	.01084	.06564	1.000	-.3196	.3413
	TGF-β1-HSC-MVs-H2O2	-.04926	.05375	.971	-.5097	.4112
HSC-MVs-H2O2	Control	-.12907	.06487	.539	-.4537	.1956
	H2O2	-.01084	.06564	1.000	-.3413	.3196
	TGF-β1-HSC-MVs-H2O2	-.06010	.04281	.851	-.3880	.2678
TGF-β1-HSC-MVs-H2O2	Control	-.06897	.05281	.888	-.5181	.3801
	H2O2	.04926	.05375	.971	-.4112	.5097
	HSC-MVs-H2O2	.06010	.04281	.851	-.2678	.3880

4-1-2. Figure 4B P-PI3K

Descriptives

VAR00002

	N	Mean	Std. Deviation	Std. Error	95% Confidence Interval for Mean		Minimum	Maximum
					Lower Bound	Upper Bound		
Control	3	1.0000	.07125	.04114	.8230	1.1770	.92	1.06
H2O2	3	.7072	.02893	.01670	.6353	.7791	.68	.73
HSC-MVs-H2O2	3	.8336	.03880	.02240	.7372	.9300	.79	.87
TGF-β1-HSC-MVs-H2O2	3	.5984	.02893	.01670	.5265	.6703	.57	.63
Total	12	.7848	.16093	.04646	.6825	.8871	.57	1.06

ANOVA

VAR00002

	Sum of Squares	df	Mean Square	F	Sig.
Between Groups	.268	3	.089	43.343	.000
Within Groups	.017	8	.002		
Total	.285	11			

Post Hoc Tests

Multiple Comparisons

Dependent Variable: VAR00002

LSD

(I) VAR00001	(J) VAR00001	Mean Difference (I-J)	Std. Error	Sig.	95% Confidence Interval	
					Lower Bound	Upper Bound
Control	H2O2	.29280*	.03709	.000	.2073	.3783
	HSC-MVs-H2O2	.16640*	.03709	.002	.0809	.2519
	TGF-β1-HSC-MVs-H2O2	.40160*	.03709	.000	.3161	.4871
H2O2	Control	-.29280*	.03709	.000	-.3783	-.2073
	HSC-MVs-H2O2	-.12640*	.03709	.009	-.2119	-.0409
	TGF-β1-HSC-MVs-H2O2	.10880*	.03709	.019	.0233	.1943
HSC-MVs-H2O2	Control	-.16640*	.03709	.002	-.2519	-.0809
	H2O2	.12640*	.03709	.009	.0409	.2119
	TGF-β1-HSC-MVs-H2O2	.23520*	.03709	.000	.1497	.3207
TGF-β1-HSC-MVs-H2O2	Control	-.40160*	.03709	.000	-.4871	-.3161
	H2O2	-.10880*	.03709	.019	-.1943	-.0233
	HSC-MVs-H2O2	-.23520*	.03709	.000	-.3207	-.1497

*. The mean difference is significant at the 0.05 level.

4-1-3. Figure 4B AKT

Descriptives

VAR00002

	N	Mean	Std. Deviation	Std. Error	95% Confidence Interval for Mean Lower Bound	95% Confidence Interval for Mean Upper Bound	Minimum	Maximum
Control	3	1.0000	.08103	.04679	.7987	1.2013	.92	1.08
H2O2	3	.9522	.07140	.04122	.7748	1.1296	.87	1.01
HSC-MVs-H2O2	3	.9615	.07286	.04206	.7805	1.1425	.88	1.01
TGF-β1-HSC-MVs-H2O2	3	1.0563	.07596	.04386	.8676	1.2450	.97	1.11
Total	12	.9925	.07723	.02229	.9434	1.0416	.87	1.11

ANOVA

VAR00002

	Sum of Squares	df	Mean Square	F	Sig.
Between Groups	.020	3	.007	1.179	.377
Within Groups	.045	8	.006		
Total	.066	11			

Post Hoc Tests

Multiple Comparisons

Dependent Variable: VAR00002

Tamhane

(I) VAR00001	(J) VAR00001	Mean Difference (I-J)	Std. Error	Sig.	95% Confidence Interval Lower Bound	95% Confidence Interval Upper Bound
Control	H2O2	.04780	.06235	.982	-.2563	.3519
	HSC-MVs-H2O2	.03848	.06291	.994	-.2673	.3443
	TGF-β1-HSC-MVs-H2O2	-.05626	.06413	.966	-.3664	.2539
H2O2	Control	-.04780	.06235	.982	-.3519	.2563
	HSC-MVs-H2O2	-.00932	.05889	1.000	-.2934	.2747
	TGF-β1-HSC-MVs-H2O2	-.10406	.06019	.647	-.3951	.1869
HSC-MVs-H2O2	Control	-.03848	.06291	.994	-.3443	.2673
	H2O2	.00932	.05889	1.000	-.2747	.2934
	TGF-β1-HSC-MVs-H2O2	-.09474	.06077	.726	-.3881	.1986
TGF-β1-HSC-MVs-H2O2	Control	.05626	.06413	.966	-.2539	.3664
	H2O2	.10406	.06019	.647	-.1869	.3951
	HSC-MVs-H2O2	.09474	.06077	.726	-.1986	.3881

4-1-4. Figure 4B P-AKT

Descriptives

VAR00002

	N	Mean	Std. Deviation	Std. Error	95% Confidence Interval for Mean		Minimum	Maximum
					Lower Bound	Upper Bound		
Control	3	1.0000	.08377	.04837	.7919	1.2081	.92	1.09
H2O2	3	.6953	.02814	.01625	.6254	.7652	.67	.72
HSC-MVs-H2O2	3	.8269	.05921	.03419	.6798	.9740	.76	.87
TGF-β1-HSC-MVs-H2O2	3	.5036	.08630	.04983	.2892	.7180	.41	.58
Total	12	.7564	.19850	.05730	.6303	.8826	.41	1.09

ANOVA

VAR00002

	Sum of Squares	df	Mean Square	F	Sig.
Between Groups	.396	3	.132	28.132	.000
Within Groups	.038	8	.005		
Total	.433	11			

Post Hoc Tests

Multiple Comparisons

Dependent Variable: VAR00002

LSD

(I) VAR00001	(J) VAR00001	Mean Difference (I-J)	Std. Error	Sig.	95% Confidence Interval	
					Lower Bound	Upper Bound
Control	H2O2	.30472*	.05592	.001	.1758	.4337
	HSC-MVs-H2O2	.17310*	.05592	.015	.0441	.3021
	TGF-β1-HSC-MVs-H2O2	.49642*	.05592	.000	.3675	.6254
H2O2	Control	-.30472*	.05592	.001	-.4337	-.1758
	HSC-MVs-H2O2	-.13162*	.05592	.046	-.2606	-.0027
	TGF-β1-HSC-MVs-H2O2	.19170*	.05592	.009	.0627	.3207
HSC-MVs-H2O2	Control	-.17310*	.05592	.015	-.3021	-.0441
	H2O2	.13162*	.05592	.046	.0027	.2606
	TGF-β1-HSC-MVs-H2O2	.32332*	.05592	.000	.1944	.4523
TGF-β1-HSC-MVs-H2O2	Control	-.49642*	.05592	.000	-.6254	-.3675
	H2O2	-.19170*	.05592	.009	-.3207	-.0627
	HSC-MVs-H2O2	-.32332*	.05592	.000	-.4523	-.1944

*. The mean difference is significant at the 0.05 level.

4-2. Figure 4D

D

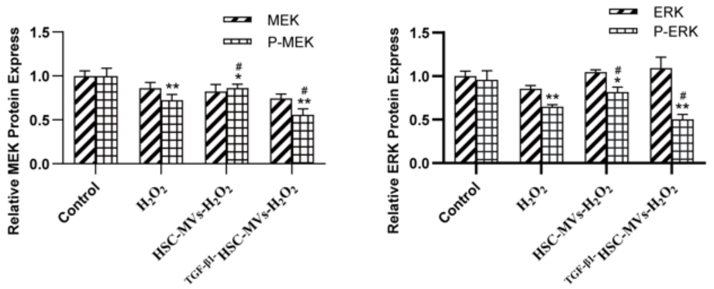

4-2-1. Figure 4D MEK

Descriptives

VAR00002

	N	Mean	Std. Deviation	Std. Error	95% Confidence Interval for Mean Lower Bound	95% Confidence Interval for Mean Upper Bound	Minimum	Maximum
Control	3	1.0000	.05629	.03250	.8602	1.1398	.94	1.06
H2O2	3	.8606	.06593	.03806	.6968	1.0243	.79	.92
HSC-MVs-H2O2	3	.8230	.07933	.04580	.6259	1.0200	.73	.87
TGF-β1-HSC-MVs-H2O2	3	.7442	.04936	.02850	.6216	.8668	.69	.79
Total	12	.8569	.11100	.03204	.7864	.9275	.69	1.06

ANOVA

VAR00002

	Sum of Squares	df	Mean Square	F	Sig.
Between Groups	.103	3	.034	8.459	.007
Within Groups	.032	8	.004		
Total	.136	11			

Post Hoc Tests

Multiple Comparisons

Dependent Variable: VAR00002

LSD

(I) VAR00001	(J) VAR00001	Mean Difference (I-J)	Std. Error	Sig.	95% Confidence Interval Lower Bound	95% Confidence Interval Upper Bound
Control	H2O2	.13942*	.05203	.028	.0194	.2594
	HSC-MVs-H2O2	.17705*	.05203	.009	.0571	.2970
	TGF-β1-HSC-MVs-H2O2	.25583*	.05203	.001	.1358	.3758
H2O2	Control	-.13942*	.05203	.028	-.2594	-.0194
	HSC-MVs-H2O2	.03762	.05203	.490	-.0824	.1576
	TGF-β1-HSC-MVs-H2O2	.11640	.05203	.056	-.0036	.2364
HSC-MVs-H2O2	Control	-.17705*	.05203	.009	-.2970	-.0571
	H2O2	-.03762	.05203	.490	-.1576	.0824
	TGF-β1-HSC-MVs-H2O2	.07878	.05203	.168	-.0412	.1988
TGF-β1-HSC-MVs-H2O2	Control	-.25583*	.05203	.001	-.3758	-.1358
	H2O2	-.11640	.05203	.056	-.2364	.0036
	HSC-MVs-H2O2	-.07878	.05203	.168	-.1988	.0412

*. The mean difference is significant at the 0.05 level.

4-2-2. Figure 4D P-MEK

Descriptives

VAR00002

	N	Mean	Std. Deviation	Std. Error	95% Confidence Interval for Mean		Minimum	Maximum
					Lower Bound	Upper Bound		
Control	3	1.0000	.08833	.05100	.7806	1.2194	.90	1.07
H2O2	3	.7236	.06590	.03805	.5599	.8873	.66	.79
HSC-MVs-H2O2	3	.8626	.04274	.02467	.7565	.9688	.82	.90
TGF-β1-HSC-MVs-H2O2	3	.5561	.06933	.04003	.3839	.7283	.48	.61
Total	12	.7856	.18161	.05242	.6702	.9010	.48	1.07

ANOVA

VAR00002

	Sum of Squares	df	Mean Square	F	Sig.
Between Groups	.325	3	.108	23.092	.000
Within Groups	.038	8	.005		
Total	.363	11			

Post Hoc Tests

Multiple Comparisons

Dependent Variable: VAR00002
LSD

(I) VAR00001	(J) VAR00001	Mean Difference (I-J)	Std. Error	Sig.	95% Confidence Interval	
					Lower Bound	Upper Bound
Control	H2O2	.27638*	.05594	.001	.1474	.4054
	HSC-MVs-H2O2	.13735*	.05594	.040	.0083	.2664
	TGF-β1-HSC-MVs-H2O2	.44389*	.05594	.000	.3149	.5729
H2O2	Control	-.27638*	.05594	.001	-.4054	-.1474
	HSC-MVs-H2O2	-.13903*	.05594	.038	-.2680	-.0100
	TGF-β1-HSC-MVs-H2O2	.16750*	.05594	.017	.0385	.2965
HSC-MVs-H2O2	Control	-.13735*	.05594	.040	-.2664	-.0083
	H2O2	.13903*	.05594	.038	.0100	.2680
	TGF-β1-HSC-MVs-H2O2	.30653*	.05594	.001	.1775	.4355
TGF-β1-HSC-MVs-H2O2	Control	-.44389*	.05594	.000	-.5729	-.3149
	H2O2	-.16750*	.05594	.017	-.2965	-.0385
	HSC-MVs-H2O2	-.30653*	.05594	.001	-.4355	-.1775

*. The mean difference is significant at the 0.05 level.

4-2-3. Figure 4D ERK

Descriptives

VAR00002

	N	Mean	Std. Deviation	Std. Error	95% Confidence Interval for Mean		Minimum	Maximum
					Lower Bound	Upper Bound		
Control	3	1.0000	.05731	.03309	.8576	1.1424	.96	1.06
H2O2	3	.8554	.03567	.02059	.7668	.9440	.81	.88
HSC-MVs-H2O2	3	1.0459	.02574	.01486	.9820	1.1099	1.02	1.07
TGF-β1-HSC-MVs-H2O2	3	1.0938	.12408	.07164	.7856	1.4020	.95	1.17
Total	12	.9988	.11145	.03217	.9280	1.0696	.81	1.17

ANOVA

VAR00002

	Sum of Squares	df	Mean Square	F	Sig.
Between Groups	.095	3	.032	6.171	.018
Within Groups	.041	8	.005		
Total	.137	11			

Post Hoc Tests

Multiple Comparisons

Dependent Variable: VAR00002

LSD

(I) VAR00001	(J) VAR00001	Mean Difference (I-J)	Std. Error	Sig.	95% Confidence Interval	
					Lower Bound	Upper Bound
Control	H2O2	.14456*	.05862	.039	.0094	.2797
	HSC-MVs-H2O2	-.04595	.05862	.456	-.1811	.0892
	TGF-β1-HSC-MVs-H2O2	-.09380	.05862	.148	-.2290	.0414
H2O2	Control	-.14456*	.05862	.039	-.2797	-.0094
	HSC-MVs-H2O2	-.19051*	.05862	.012	-.3257	-.0553
	TGF-β1-HSC-MVs-H2O2	-.23836*	.05862	.004	-.3735	-.1032
HSC-MVs-H2O2	Control	.04595	.05862	.456	-.0892	.1811
	H2O2	.19051*	.05862	.012	.0553	.3257
	TGF-β1-HSC-MVs-H2O2	-.04785	.05862	.438	-.1830	.0873
TGF-β1-HSC-MVs-H2O2	Control	.09380	.05862	.148	-.0414	.2290
	H2O2	.23836*	.05862	.004	.1032	.3735
	HSC-MVs-H2O2	.04785	.05862	.438	-.0873	.1830

*. The mean difference is significant at the 0.05 level.

4-2-4. Figure 4D P-ERK

Descriptives

VAR00002

	N	Mean	Std. Deviation	Std. Error	95% Confidence Interval for Mean		Minimum	Maximum
					Lower Bound	Upper Bound		
Control	3	.9572	.10518	.06073	.6959	1.2184	.85	1.06
H2O2	3	.6486	.02360	.01363	.5899	.7072	.63	.67
HSC-MVs-H2O2	3	.8172	.05593	.03229	.6782	.9561	.75	.86
TGF-β1-HSC-MVs-H2O2	3	.5043	.05444	.03143	.3691	.6395	.44	.54
Total	12	.7318	.18725	.05405	.6128	.8508	.44	1.06

ANOVA

VAR00002

	Sum of Squares	df	Mean Square	F	Sig.
Between Groups	.350	3	.117	26.367	.000
Within Groups	.035	8	.004		
Total	.386	11			

Post Hoc Tests

Multiple Comparisons

Dependent Variable: VAR00002

LSD

(I) VAR00001	(J) VAR00001	Mean Difference (I-J)	Std. Error	Sig.	95% Confidence Interval	
					Lower Bound	Upper Bound
Control	H2O2	.30858*	.05433	.000	.1833	.4339
	HSC-MVs-H2O2	.14000*	.05433	.033	.0147	.2653
	TGF-β1-HSC-MVs-H2O2	.45286*	.05433	.000	.3276	.5782
H2O2	Control	-.30858*	.05433	.000	-.4339	-.1833
	HSC-MVs-H2O2	-.16857*	.05433	.015	-.2939	-.0433
	TGF-β1-HSC-MVs-H2O2	.14429*	.05433	.029	.0190	.2696
HSC-MVs-H2O2	Control	-.14000*	.05433	.033	-.2653	-.0147
	H2O2	.16857*	.05433	.015	.0433	.2939
	TGF-β1-HSC-MVs-H2O2	.31286*	.05433	.000	.1876	.4382
TGF-β1-HSC-MVs-H2O2	Control	-.45286*	.05433	.000	-.5782	-.3276
	H2O2	-.14429*	.05433	.029	-.2696	-.0190
	HSC-MVs-H2O2	-.31286*	.05433	.000	-.4382	-.1876

*. The mean difference is significant at the 0.05 level.

4-3. Figure 4F

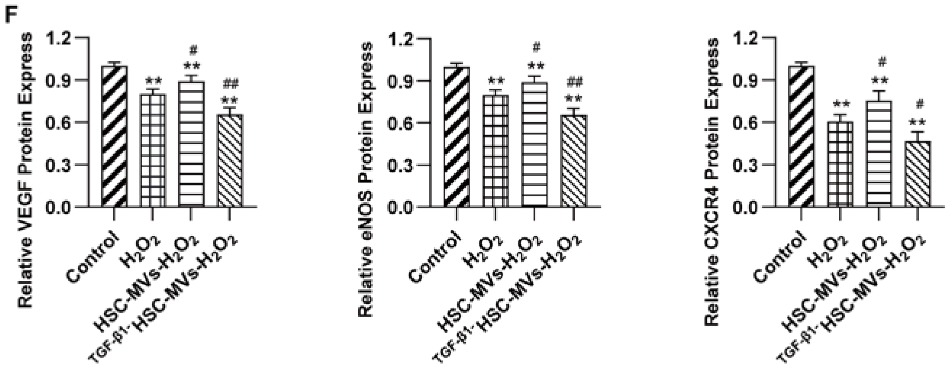

4-3-1. Figure 4F VEGF

Descriptives

VAR00002

	N	Mean	Std. Deviation	Std. Error	95% Confidence Interval for Mean		Minimum	Maximum
					Lower Bound	Upper Bound		
Control	3	1.0000	.02325	.01342	.9423	1.0578	.98	1.03
H2O2	3	.7980	.03659	.02113	.7071	.8889	.76	.83
HSC-MVs-H2O2	3	.8906	.04204	.02427	.7862	.9950	.85	.94
TGF-β1-HSC-MVs-H2O2	3	.6582	.04469	.02580	.5472	.7692	.61	.70
Total	12	.8367	.13488	.03894	.7510	.9224	.61	1.03

ANOVA

VAR00002

	Sum of Squares	df	Mean Square	F	Sig.
Between Groups	.189	3	.063	44.610	.000
Within Groups	.011	8	.001		
Total	.200	11			

Post Hoc Tests

Multiple Comparisons

Dependent Variable: VAR00002
LSD

(I) VAR00001	(J) VAR00001	Mean Difference (I-J)	Std. Error	Sig.	95% Confidence Interval	
					Lower Bound	Upper Bound
Control	H2O2	.20205*	.03067	.000	.1313	.2728
	HSC-MVs-H2O2	.10941*	.03067	.007	.0387	.1801
	TGF-β1-HSC-MVs-H2O2	.34182*	.03067	.000	.2711	.4125
H2O2	Control	-.20205*	.03067	.000	-.2728	-.1313
	HSC-MVs-H2O2	-.09264*	.03067	.017	-.1634	-.0219
	TGF-β1-HSC-MVs-H2O2	.13977*	.03067	.002	.0690	.2105
HSC-MVs-H2O2	Control	-.10941*	.03067	.007	-.1801	-.0387
	H2O2	.09264*	.03067	.017	.0219	.1634
	TGF-β1-HSC-MVs-H2O2	.23241*	.03067	.000	.1617	.3031
TGF-β1-HSC-MVs-H2O2	Control	-.34182*	.03067	.000	-.4125	-.2711
	H2O2	-.13977*	.03067	.002	-.2105	-.0690
	HSC-MVs-H2O2	-.23241*	.03067	.000	-.3031	-.1617

*. The mean difference is significant at the 0.05 level.

4-3-2. Figure 4F eNOS

Descriptives

VAR00002

	N	Mean	Std. Deviation	Std. Error	95% Confidence Interval for Mean		Minimum	Maximum
					Lower Bound	Upper Bound		
Control	3	1.0000	.02325	.01342	.9423	1.0578	.98	1.03
H2O2	3	.7980	.03659	.02113	.7071	.8889	.76	.83
HSC-MVs-H2O2	3	.8906	.04204	.02427	.7862	.9950	.85	.94
TGF-β1-HSC-MVs-H2O2	3	.6582	.04469	.02580	.5472	.7692	.61	.70
Total	12	.8367	.13488	.03894	.7510	.9224	.61	1.03

ANOVA

VAR00002

	Sum of Squares	df	Mean Square	F	Sig.
Between Groups	.189	3	.063	44.610	.000
Within Groups	.011	8	.001		
Total	.200	11			

Post Hoc Tests

Multiple Comparisons

Dependent Variable: VAR00002
LSD

(I) VAR00001	(J) VAR00001	Mean Difference (I-J)	Std. Error	Sig.	95% Confidence Interval	
					Lower Bound	Upper Bound
Control	H2O2	.20205*	.03067	.000	.1313	.2728
	HSC-MVs-H2O2	.10941*	.03067	.007	.0387	.1801
	TGF-β1-HSC-MVs-H2O2	.34182*	.03067	.000	.2711	.4125
H2O2	Control	-.20205*	.03067	.000	-.2728	-.1313
	HSC-MVs-H2O2	-.09264*	.03067	.017	-.1634	-.0219
	TGF-β1-HSC-MVs-H2O2	.13977*	.03067	.002	.0690	.2105
HSC-MVs-H2O2	Control	-.10941*	.03067	.007	-.1801	-.0387
	H2O2	.09264*	.03067	.017	.0219	.1634
	TGF-β1-HSC-MVs-H2O2	.23241*	.03067	.000	.1617	.3031
TGF-β1-HSC-MVs-H2O2	Control	-.34182*	.03067	.000	-.4125	-.2711
	H2O2	-.13977*	.03067	.002	-.2105	-.0690
	HSC-MVs-H2O2	-.23241*	.03067	.000	-.3031	-.1617

*. The mean difference is significant at the 0.05 level.

4-3-3. Figure 4F CXCR4

Descriptives

VAR00002

	N	Mean	Std. Deviation	Std. Error	95% Confidence Interval for Mean		Minimum	Maximum
					Lower Bound	Upper Bound		
Control	3	1.0000	.02325	.01342	.9423	1.0578	.98	1.03
H2O2	3	.6052	.05109	.02950	.4783	.7321	.55	.65
HSC-MVs-H2O2	3	.7535	.06802	.03927	.5846	.9225	.70	.83
TGF-β1-HSC-MVs-H2O2	3	.4676	.06741	.03892	.3002	.6351	.40	.53
Total	12	.7066	.21143	.06104	.5723	.8409	.40	1.03

ANOVA

VAR00002

	Sum of Squares	df	Mean Square	F	Sig.
Between Groups	.467	3	.156	50.542	.000
Within Groups	.025	8	.003		
Total	.492	11			

Post Hoc Tests

Multiple Comparisons

Dependent Variable: VAR00002

LSD

(I) VAR00001	(J) VAR00001	Mean Difference (I-J)	Std. Error	Sig.	95% Confidence Interval	
					Lower Bound	Upper Bound
Control	H2O2	.39482*	.04532	.000	.2903	.4993
	HSC-MVs-H2O2	.24648*	.04532	.001	.1420	.3510
	TGF-β1-HSC-MVs-H2O2	.53241*	.04532	.000	.4279	.6369
H2O2	Control	-.39482*	.04532	.000	-.4993	-.2903
	HSC-MVs-H2O2	-.14834*	.04532	.011	-.2528	-.0438
	TGF-β1-HSC-MVs-H2O2	.13760*	.04532	.016	.0331	.2421
HSC-MVs-H2O2	Control	-.24648*	.04532	.001	-.3510	-.1420
	H2O2	.14834*	.04532	.011	.0438	.2528
	TGF-β1-HSC-MVs-H2O2	.28593*	.04532	.000	.1814	.3904
TGF-β1-HSC-MVs-H2O2	Control	-.53241*	.04532	.000	-.6369	-.4279
	H2O2	-.13760*	.04532	.016	-.2421	-.0331
	HSC-MVs-H2O2	-.28593*	.04532	.000	-.3904	-.1814

*. The mean difference is significant at the 0.05 level.

5. Figure 5

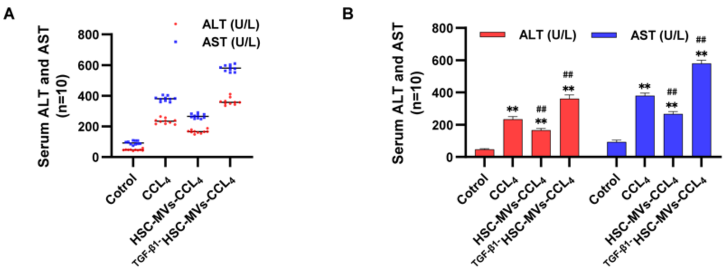

5-1. Figure 5 ALT

Descriptives

VAR00002

	N	Mean	Std. Deviation	Std. Error	95% Confidence Interval for Mean		Minimum	Maximum
					Lower Bound	Upper Bound		
Control	10	46.6400	4.69496	1.48468	43.2814	49.9986	40.00	57.90
H2O2	10	233.6100	17.28683	5.46657	221.2438	245.9762	212.30	264.80
HSC-MVs-H2O2	10	165.6900	11.30717	3.57564	157.6013	173.7787	147.60	187.60
TGF-β1-HSC-MVs-H2O2	10	361.7200	22.88249	7.23608	345.3509	378.0891	334.70	410.80
Total	40	201.9150	116.39884	18.40427	164.6888	239.1412	40.00	410.80

ANOVA

VAR00002

	Sum of Squares	df	Mean Square	F	Sig.
Between Groups	519647.873	3	173215.958	712.575	.000
Within Groups	8751.038	36	243.084		
Total	528398.911	39			

Post Hoc Tests

Multiple Comparisons

Dependent Variable: VAR00002

LSD

(I) VAR00001	(J) VAR00001	Mean Difference (I-J)	Std. Error	Sig.	95% Confidence Interval	
					Lower Bound	Upper Bound
Control	H2O2	-186.97000*	6.97258	.000	-201.1110	-172.8290
	HSC-MVs-H2O2	-119.05000*	6.97258	.000	-133.1910	-104.9090
	TGF-β1-HSC-MVs-H2O2	-315.08000*	6.97258	.000	-329.2210	-300.9390
H2O2	Control	186.97000*	6.97258	.000	172.8290	201.1110
	HSC-MVs-H2O2	67.92000*	6.97258	.000	53.7790	82.0610
	TGF-β1-HSC-MVs-H2O2	-128.11000*	6.97258	.000	-142.2510	-113.9690
HSC-MVs-H2O2	Control	119.05000*	6.97258	.000	104.9090	133.1910
	H2O2	-67.92000*	6.97258	.000	-82.0610	-53.7790
	TGF-β1-HSC-MVs-H2O2	-196.03000*	6.97258	.000	-210.1710	-181.8890
TGF-β1-HSC-MVs-H2O2	Control	315.08000*	6.97258	.000	300.9390	329.2210
	H2O2	128.11000*	6.97258	.000	113.9690	142.2510
	HSC-MVs-H2O2	196.03000*	6.97258	.000	181.8890	210.1710

*. The mean difference is significant at the 0.05 level.

5-2. Figure 5 AST

Descriptives

VAR00002

	N	Mean	Std. Deviation	Std. Error	95% Confidence Interval for Mean		Minimum	Maximum
					Lower Bound	Upper Bound		
Control	10	93.6000	11.66705	3.68944	85.2539	101.9461	75.30	108.70
H2O2	10	380.2100	15.91725	5.03348	368.8235	391.5965	357.80	405.70
HSC-MVs-H2O2	10	266.5400	14.36788	4.54352	256.2618	276.8182	248.30	290.40
TGF-β1-HSC-MVs-H2O2	10	579.4500	20.57141	6.50525	564.7341	594.1659	550.80	610.50
Total	40	329.9500	179.44048	28.37203	272.5622	387.3378	75.30	610.50

ANOVA

VAR00002

	Sum of Squares	df	Mean Square	F	Sig.
Between Groups	1246584.682	3	415528.227	1630.965	.000
Within Groups	9171.878	36	254.774		
Total	1255756.560	39			

Post Hoc Tests

Multiple Comparisons

Dependent Variable: VAR00002

LSD

(I) VAR00001	(J) VAR00001	Mean Difference (I-J)	Std. Error	Sig.	95% Confidence Interval	
					Lower Bound	Upper Bound
Control	H2O2	-286.61000*	7.13827	.000	-301.0871	-272.1329
	HSC-MVs-H2O2	-172.94000*	7.13827	.000	-187.4171	-158.4629
	TGF-β1-HSC-MVs-H2O2	-485.85000*	7.13827	.000	-500.3271	-471.3729
H2O2	Control	286.61000*	7.13827	.000	272.1329	301.0871
	HSC-MVs-H2O2	113.67000*	7.13827	.000	99.1929	128.1471
	TGF-β1-HSC-MVs-H2O2	-199.24000*	7.13827	.000	-213.7171	-184.7629
HSC-MVs-H2O2	Control	172.94000*	7.13827	.000	158.4629	187.4171
	H2O2	-113.67000*	7.13827	.000	-128.1471	-99.1929
	TGF-β1-HSC-MVs-H2O2	-312.91000*	7.13827	.000	-327.3871	-298.4329
TGF-β1-HSC-MVs-H2O2	Control	485.85000*	7.13827	.000	471.3729	500.3271
	H2O2	199.24000*	7.13827	.000	184.7629	213.7171
	HSC-MVs-H2O2	312.91000*	7.13827	.000	298.4329	327.3871

*. The mean difference is significant at the 0.05 level.

6. Figure 6

6-1. Figure 6 E

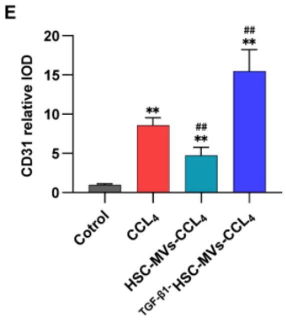

Descriptives

VAR00002

	N	Mean	Std. Deviation	Std. Error	95% Confidence Interval for Mean Lower Bound	Upper Bound	Minimum	Maximum
Control	5	1.0002	.12616	.05642	.8435	1.1568	.82	1.13
CCL4	5	8.5635	.98136	.43888	7.3450	9.7821	7.41	9.72
HSC-MVs-CCL4	5	4.7394	1.04704	.46825	3.4393	6.0395	3.62	5.79
TGF-β1-HSC-MVs-CCL4	5	15.4561	2.74661	1.22832	12.0457	18.8665	12.55	18.37
Total	20	7.4398	5.66568	1.26688	4.7882	10.0914	.82	18.37

ANOVA

VAR00002

	Sum of Squares	df	Mean Square	F	Sig.
Between Groups	571.422	3	190.474	79.206	.000
Within Groups	38.476	16	2.405		
Total	609.898	19			

Post Hoc Tests

Multiple Comparisons

Dependent Variable: VAR00002

LSD

(I) VAR00001	(J) VAR00001	Mean Difference (I-J)	Std. Error	Sig.	95% Confidence Interval Lower Bound	Upper Bound
Control	CCL4	-7.56335*	.98077	.000	-9.6425	-5.4842
	HSC-MVs-CCL4	-3.73923*	.98077	.002	-5.8184	-1.6601
	TGF-β1-HSC-MVs-CCL4	-14.45590*	.98077	.000	-16.5350	-12.3768
CCL4	Control	7.56335*	.98077	.000	5.4842	9.6425
	HSC-MVs-CCL4	3.82412*	.98077	.001	1.7450	5.9033
	TGF-β1-HSC-MVs-CCL4	-6.89255*	.98077	.000	-8.9717	-4.8134
HSC-MVs-CCL4	Control	3.73923*	.98077	.002	1.6601	5.8184
	CCL4	-3.82412*	.98077	.001	-5.9033	-1.7450
	TGF-β1-HSC-MVs-CCL4	-10.71667*	.98077	.000	-12.7958	-8.6375
TGF-β1-HSC-MVs-CCL4	Control	14.45590*	.98077	.000	12.3768	16.5350
	CCL4	6.89255*	.98077	.000	4.8134	8.9717
	HSC-MVs-CCL4	10.71667*	.98077	.000	8.6375	12.7958

*. The mean difference is significant at the 0.05 level.

6-2. Figure 6 F

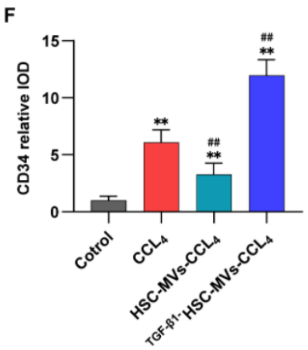

Descriptives

VAR00002

	N	Mean	Std. Deviation	Std. Error	95% Confidence Interval for Mean Lower Bound	Upper Bound	Minimum	Maximum
Control	5	1.0001	.36218	.16197	.5504	1.4498	.56	1.32
CCL4	5	6.0962	1.09882	.49141	4.7318	7.4606	4.61	7.35
HSC-MVs-CCL4	5	3.2789	.98302	.43962	2.0584	4.4995	1.71	4.19
TGF-β1-HSC-MVs-CCL4	5	11.9671	1.37912	.61676	10.2547	13.6795	10.39	13.80
Total	20	5.5856	4.31332	.96449	3.5669	7.6043	.56	13.80

ANOVA

VAR00002

	Sum of Squares	df	Mean Square	F	Sig.
Between Groups	336.662	3	112.221	106.702	.000
Within Groups	16.827	16	1.052		
Total	353.490	19			

Post Hoc Tests

Multiple Comparisons

Dependent Variable: VAR00002

LSD

(I) VAR00001	(J) VAR00001	Mean Difference (I-J)	Std. Error	Sig.	95% Confidence Interval Lower Bound	Upper Bound
Control	CCL4	-5.09614*	.64860	.000	-6.4711	-3.7212
	HSC-MVs-CCL4	-2.27888*	.64860	.003	-3.6539	-.9039
	TGF-β1-HSC-MVs-CCL4	-10.96706*	.64860	.000	-12.3420	-9.5921
CCL4	Control	5.09614*	.64860	.000	3.7212	6.4711
	HSC-MVs-CCL4	2.81726*	.64860	.001	1.4423	4.1922
	TGF-β1-HSC-MVs-CCL4	-5.87093*	.64860	.000	-7.2459	-4.4959
HSC-MVs-CCL4	Control	2.27888*	.64860	.003	.9039	3.6539
	CCL4	-2.81726*	.64860	.001	-4.1922	-1.4423
	TGF-β1-HSC-MVs-CCL4	-8.68819*	.64860	.000	-10.0632	-7.3132
TGF-β1-HSC-MVs-CCL4	Control	10.96706*	.64860	.000	9.5921	12.3420
	CCL4	5.87093*	.64860	.000	4.4959	7.2459
	HSC-MVs-CCL4	8.68819*	.64860	.000	7.3132	10.0632

*. The mean difference is significant at the 0.05 level.

6-3. Figure 6 G

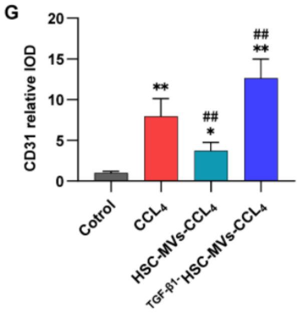

Descriptives

VAR00002

	N	Mean	Std. Deviation	Std. Error	95% Confidence Interval for Mean		Minimum	Maximum
					Lower Bound	Upper Bound		
Control	5	1.0000	.20979	.09382	.7395	1.2605	.78	1.28
CCL4	5	7.9437	2.19587	.98202	5.2172	10.6703	5.20	10.50
HSC-MVs-CCL4	5	3.7240	1.01641	.45455	2.4620	4.9861	2.76	5.20
TGF-β1-HSC-MVs-CCL4	5	12.6375	2.32606	1.04025	9.7493	15.5257	9.63	15.94
Total	20	6.3263	4.77483	1.06768	4.0916	8.5610	.78	15.94

ANOVA

VAR00002

	Sum of Squares	df	Mean Square	F	Sig.
Between Groups	387.943	3	129.314	45.737	.000
Within Groups	45.238	16	2.827		
Total	433.181	19			

Post Hoc Tests

Multiple Comparisons

Dependent Variable: VAR00002
LSD

(I) VAR00001	(J) VAR00001	Mean Difference (I-J)	Std. Error	Sig.	95% Confidence Interval	
					Lower Bound	Upper Bound
Control	CCL4	-6.94375*	1.06346	.000	-9.1982	-4.6893
	HSC-MVs-CCL4	-2.72404*	1.06346	.021	-4.9785	-.4696
	TGF-β1-HSC-MVs-CCL4	-11.63750*	1.06346	.000	-13.8919	-9.3831
CCL4	Control	6.94375*	1.06346	.000	4.6893	9.1982
	HSC-MVs-CCL4	4.21971*	1.06346	.001	1.9653	6.4741
	TGF-β1-HSC-MVs-CCL4	-4.69375*	1.06346	.000	-6.9482	-2.4393
HSC-MVs-CCL4	Control	2.72404*	1.06346	.021	.4696	4.9785
	CCL4	-4.21971*	1.06346	.001	-6.4741	-1.9653
	TGF-β1-HSC-MVs-CCL4	-8.91346*	1.06346	.000	-11.1679	-6.6590
TGF-β1-HSC-MVs-CCL4	Control	11.63750*	1.06346	.000	9.3831	13.8919
	CCL4	4.69375*	1.06346	.000	2.4393	6.9482
	HSC-MVs-CCL4	8.91346*	1.06346	.000	6.6590	11.1679

*. The mean difference is significant at the 0.05 level.

6-4. Figure 6 H

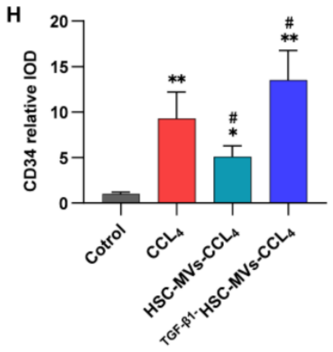

Descriptives

VAR00002

	N	Mean	Std. Deviation	Std. Error	95% Confidence Interval for Mean		Minimum	Maximum
					Lower Bound	Upper Bound		
Control	5	1.0000	.19071	.08529	.7632	1.2368	.70	1.22
CCL4	5	9.2870	2.92966	1.31018	5.6494	12.9247	5.15	13.32
HSC-MVs-CCL4	5	5.0815	1.20552	.53913	3.5846	6.5783	3.49	6.34
TGF-β1-HSC-MVs-CCL4	5	13.4930	3.28696	1.46997	9.4117	17.5743	10.20	18.04
Total	20	7.2154	5.22105	1.16746	4.7718	9.6589	.70	18.04

ANOVA

VAR00002

	Sum of Squares	df	Mean Square	F	Sig.
Between Groups	434.422	3	144.807	27.745	.000
Within Groups	83.507	16	5.219		
Total	517.928	19			

Post Hoc Tests

Multiple Comparisons

Dependent Variable: VAR00002

LSD

(I) VAR00001	(J) VAR00001	Mean Difference (I-J)	Std. Error	Sig.	95% Confidence Interval	
					Lower Bound	Upper Bound
Control	CCL4	-8.28703*	1.44488	.000	-11.3500	-5.2240
	HSC-MVs-CCL4	-4.08149*	1.44488	.012	-7.1445	-1.0185
	TGF-β1-HSC-MVs-CCL4	-12.49297*	1.44488	.000	-15.5560	-9.4300
CCL4	Control	8.28703*	1.44488	.000	5.2240	11.3500
	HSC-MVs-CCL4	4.20554*	1.44488	.010	1.1425	7.2685
	TGF-β1-HSC-MVs-CCL4	-4.20594*	1.44488	.010	-7.2689	-1.1429
HSC-MVs-CCL4	Control	4.08149*	1.44488	.012	1.0185	7.1445
	CCL4	-4.20554*	1.44488	.010	-7.2685	-1.1425
	TGF-β1-HSC-MVs-CCL4	-8.41148*	1.44488	.000	-11.4745	-5.3485
TGF-β1-HSC-MVs-CCL4	Control	12.49297*	1.44488	.000	9.4300	15.5560
	CCL4	4.20594*	1.44488	.010	1.1429	7.2689
	HSC-MVs-CCL4	8.41148*	1.44488	.000	5.3485	11.4745

*. The mean difference is significant at the 0.05 level.

7. Figure 7 B

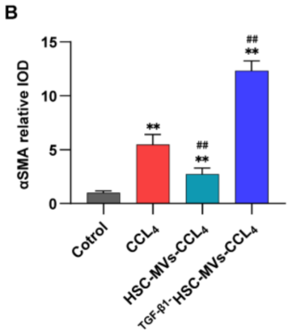

Descriptives

VAR00002

	N	Mean	Std. Deviation	Std. Error	95% Confidence Interval for Mean Lower Bound	95% Confidence Interval for Mean Upper Bound	Minimum	Maximum
Control	5	1.0002	.16507	.07382	.7953	1.2052	.74	1.19
CCL4	5	5.4713	.92488	.41362	4.3229	6.6197	4.33	6.88
HSC-MVs-CCL4	5	2.7242	.55817	.24962	2.0311	3.4173	1.89	3.45
TGF-β1-HSC-MVs-CCL4	5	12.3445	.90640	.40535	11.2191	13.4700	11.30	13.51
Total	20	5.3851	4.48274	1.00237	3.2871	7.4830	.74	13.51

ANOVA

VAR00002

	Sum of Squares	df	Mean Square	F	Sig.
Between Groups	373.741	3	124.580	247.212	.000
Within Groups	8.063	16	.504		
Total	381.804	19			

Post Hoc Tests

Multiple Comparisons

Dependent Variable: VAR00002
LSD

(I) VAR00001	(J) VAR00001	Mean Difference (I-J)	Std. Error	Sig.	95% Confidence Interval Lower Bound	95% Confidence Interval Upper Bound
Control	CCL4	-4.47102*	.44897	.000	-5.4228	-3.5192
	HSC-MVs-CCL4	-1.72396*	.44897	.001	-2.6757	-.7722
	TGF-β1-HSC-MVs-CCL4	-11.34427*	.44897	.000	-12.2960	-10.3925
CCL4	Control	4.47102*	.44897	.000	3.5192	5.4228
	HSC-MVs-CCL4	2.74706*	.44897	.000	1.7953	3.6988
	TGF-β1-HSC-MVs-CCL4	-6.87326*	.44897	.000	-7.8250	-5.9215
HSC-MVs-CCL4	Control	1.72396*	.44897	.001	.7722	2.6757
	CCL4	-2.74706*	.44897	.000	-3.6988	-1.7953
	TGF-β1-HSC-MVs-CCL4	-9.62031*	.44897	.000	-10.5721	-8.6685
TGF-β1-HSC-MVs-CCL4	Control	11.34427*	.44897	.000	10.3925	12.2960
	CCL4	6.87326*	.44897	.000	5.9215	7.8250
	HSC-MVs-CCL4	9.62031*	.44897	.000	8.6685	10.5721

*. The mean difference is significant at the 0.05 level.
